# Characterization of the *Vibrio anguillarum Va*RyhB regulon and role in pathogenesis

**DOI:** 10.3389/fcimb.2024.1531176

**Published:** 2025-01-21

**Authors:** Yingjie Li, Xinran Yu, Peng Li, Xin Li, Lushan Wang

**Affiliations:** ^1^ State Key Laboratory of Microbial Technology, Shandong University, Qingdao, China; ^2^ Research and Development Department, China Rongtong Agricultural Development Group Co., Ltd., Hangzhou, China

**Keywords:** *Vibrio anguillarum*, VaRyhB, iron homeostasis, siderophore synthesis, chemotaxis and motility, pathogenesis

## Abstract

**Background:**

The marine Gram-negative bacterium *Vibrio anguillarum* is one of the major pathogens in aquaculture. Iron uptake is a prerequisite for virulence and is strictly controlled by a global iron uptake regulator, Fur, which acts as a repressor under iron-replete conditions. When iron is depleted, Fur also functions as an activator, playing an important role in pathogenesis. It is unclear whether this upregulation model is mediated by a small RNA, RyhB.

**Methods:**

The small RNA, *VaryhB*, was deleted in *V. anguillarum* strain 775, and its regulon was investigated using transcriptomic analysis. The roles of VaRyhB in siderophore synthesis, chemotaxis and motility, and oxidative stress were evaluated using chrome azurol S (CAS) liquid assay, swimming motility assay, and intracellular reactive oxygen species (ROS) assay, respectively. The virulence of VaRyhB was evaluated by challenging turbot larvae intraperitoneally.

**Results:**

The small RNA called VaRyhB identified in *V. anguillarum* strain 775 is significantly longer than that in Escherichia coli. Transcriptomic analysis revealed that VaRyhB is critical for iron homeostasis under limited iron conditions, and deletion of VaRyhB resulted in lower expression levels of certain genes for siderophore biosynthesis and transport, thereby leading to impaired growth, reduced siderophore production, and decreased pathogenesis. The virulence factor motility is also upregulated by VaRyhB, and reduced motility capability was observed in the ΔVaryhB mutant, which may be another reason resulting in weak pathogenesis. The sensitivity toward H2O2 in the ΔVafur mutant could be restored by the loss of VaRyhB, suggesting that the role of Fur in oxidative stress is mediated by VaRyhB. VaRyhB also functions to inhibit the expression of genes involved in Fe-S assembly and the TCA cycle. In addition, two aspects of the type VI secretion system and molybdenum cofactor biosynthesis were first identified as being regulated by VaRyhB.

**Conclusion:**

In *V. anguillarum*, the sRNA VaRyhB plays a critical role in the inhibition of genes involved in the TCA cycle, Fe-S assembly, and the type VI secretion system. It is also essential for the activation of siderophore synthesis, chemotaxis and motility, and anaerobic denitrification. Our work provides the first evidence of the VaRyhB regulon and its role in the pathogenesis of *V. anguillarum*.

## 1 Introduction

The marine-derived *Vibrio anguillarum* is a common pathogenic bacterium and leads to serious vibriosis with hemorrhagic septicemia in many fish species. The *V. anguillarum* strains can be divided into more than 20 serotypes (Toranzo and Barja, 1990), and only serotypes O1, O2, and partial O3 are involved in vibriosis outbreaks (Toranzo et al., 2005). A number of virulence factors have been identified, such as extracellular metalloproteases (Norqvist et al., 1990; Yang et al., 2007; Mo et al., 2010), proteins involved in chemotaxis and motility (Milton et al., 1996; Ormonde et al., 2000), lipopolysaccharides (Welch and Crosa, 2005), hemolysins (Rodkhum et al., 2005; Rock and Nelson, 2006; Li et al., 2008; Xu et al., 2011; Mou et al., 2013), exopolysaccharides (Croxatto et al., 2007), and iron acquisition systems (Naka and Crosa, 2011). Among these factors, iron uptake is one of the key steps for bacterial infection.

*V. anguillarum* strains employ diverse iron-sequestering strategies to cope with different iron conditions, including multiple siderophore-dependent systems (Balado et al., 2006; 2008; Naka et al., 2013), the heme uptake system (Mazoy et al., 2003; Mouriño et al., 2004), ferrous iron uptake (*feoABC*), and ferric iron uptake (two *fbpABC* clusters) (Naka and Crosa, 2011; Li and Ma, 2017). Among them, siderophore-dependent systems and the heme uptake system have been reported to be associated with virulence. In siderophore-dependent systems, three different pathways are present in *V. anguillarum* strains: one is vanchrobactin-dependent, which is present in endogenous plasmidless species; one is anguibactin-dependent, which is observed in endogenous plasmid-containing species; and the third one is for the uptake of xenosiderophore ferrichrome (Li and Ma, 2017). The complex iron uptake is strictly controlled by the global iron sensor, the Ferric-Uptake Regulator (Fur). The deletion of *Va*Fur in the *V. anguillarum* strain 775 led to increased expression of genes involved in the iron uptake system under iron-replete conditions (Li et al., 2024). Loss of *Va*Fur also resulted in decreased pathogenesis, which should not be directly caused by aberrantly regulated iron uptake since free iron is limited in the host. Our previous work revealed that some critical virulence factors, including extracellular metalloprotease EmpA and proteins involved in chemotaxis and motility, are activated by *Va*Fur under limited iron conditions (Li et al., 2024). Therefore, in addition to being a repressor for iron uptake, *Va*Fur also acts as an activator for certain genes involved in virulence and oxidative stress. This model has been commonly observed in pathogenic bacteria (Porcheron and Dozois, 2015), which was first clarified in *Escherichia coli* by Massé and Gottesman (Massé and Gottesman, 2002). During this process, a small RNA (sRNA) called RyhB functions for the downregulation of certain genes when iron is depleted, and when iron is replete, the expression of RyhB is repressed by Fur (Troxell and Hassan, 2013; Chareyre and Mandin, 2018).

In this work, we aimed to uncover the role of RyhB (*Va*RyhB) in iron homeostasis and virulence in the *V. anguillarum* strain 775 isolated from the marine fish disease vibriosis (Crosa, 1980). Our study revealed that *Va*RyhB could act as a repressor for genes involved in the tricarboxylic acid (TCA) cycle, Fe-S assembly, and the type VI secretion system (T6SS). In addition, it also acts as an activator for certain genes responsible for siderophore anguibactin synthesis and transport, chemotaxis and motility, and anaerobic denitrification. This regulation is not always associated with *Va*Fur. The deletion of *Va*RyhB led to impaired growth and reduced motility capability under different iron conditions, thereby leading to decreased pathogenesis toward turbot larvae. Our work provides the first evidence for the role of *Va*RyhB in the *V. anguillarum* pathogenesis.

## 2 Materials and methods

### 2.1 Materials

PrimeSTAR^®^Max DNA polymerase, PrimeScript™ RT reagent kit, and SYBR^@^Premix Ex Taq™ II were purchased from TaKaRa (Tokyo, Japan). Restriction enzymes, T4 DNA ligase, and protein markers were obtained from Thermo Fisher Scientific (Waltham, MA, USA). Chloramphenicol, diaminopimelic acid (DAP), 2, 2’-dipyridine, chrome azurol S (CAS), agar for swimming motility assays, dimethyl sulfoxide (DMSO), and 2’,7’-dichlorodihydrofluorescein (H_2_DCFDA) were purchased from Sigma-Aldrich (St. Louis, MO, USA). All other molecular kits and DNA markers were purchased from TIANGEN (Beijing, China). If not specified, all other chemical reagents were obtained from Sangon Biotech (Shanghai, China).

### 2.2 Bacterial strains and growth conditions

*V. anguillarum* 775 (ATCC 68554) strains and *E. coli* strains used in this study are shown in **Supplementary Table S1**. *V. anguillarum* 775 cells were grown at 26°C in an M9 high salt medium (90 mM Na_2_HPO_4_, 22 mM KH_2_PO_4_, 18.8 mM NH_4_Cl, 345 mM NaCl, and 1 mM MgSO_4_) supplemented with 0.2% casamino acid. If not specified, rich iron conditions were achieved by adding 50 μM FeCl_3_, and limited iron conditions were obtained by adding 50 μM 2, 2’-dipyridine as described in our previous study (Li et al., 2024). For growth assays under different iron conditions, the inocula were grown under their respective iron conditions to the exponential phase. *E. coli* strains were incubated in lysogeny broth (LB) at 37°C. When the donor *E. coli* strain X7213 was used, 0.5% DAP was added.

### 2.3 Bioinformatic analysis

The *Va*RyhB sRNA was identified from the genome of *V. anguillarum* 775 (GenBank number of chromosome 1: CP002284.1; GenBank number of chromosome 2: CP002285.1; and GenBank number of the endogenous plasmid pJM1: AY312585.1) by using *Vibrio cholerae* RyhB as a query (Di Lorenzo et al., 2003; Naka et al., 2011). Sequence alignment was performed by ClustalW (Larkin et al., 2007).

### 2.4 Genetic and molecular biology techniques

DNA purification, digestion, ligation, and transformation were carried out based on standard molecular biological techniques (Sambrook and Russel, 2001). PCR products were sequenced by Beijing Tsingke Biotech Co., Ltd. (Qingdao, China) and analyzed by the software Vector NTI Advance 11.5.1 (Invitrogen, Darmstadt, Germany). Oligonucleotide sequences used in this study are listed in **Supplementary Table S2**.

### 2.5 Construction of deletion mutant strains

To construct the unmarked deletion mutants Δ*VaryhB* and Δ*Vafur*Δ*VaryhB*, a homologous recombination technique was carried out as previously described (Li et al., 2024). In brief, fusion PCR was performed to obtain the 2,000-bp flanking fragment of *VaryhB*, and the KpnI/SmaI-digested fragment was ligated into pRE112 to generate the plasmid pLYJ220. Then, the pLYJ220-containing donor strain *E. coli* X7213 was used to transform the plasmid into the *V. anguillarum* 775 wild-type (WT) strain and the Δ*Vafur* mutant by conjugation as follows: 500 μL of exponential phase *E. coli* X7213 cells were gently mixed with 800 μL of exponential phase *V. anguillarum* 775 strains. The mixture was collected by centrifugation at 3,500 *g* for 10 min and dropped in an LB plate in the presence of 0.5% DAP. After incubation overnight at 26°C, the mixed cells were suspended in 10 mL of LB medium, and 100 μL was plated in an LB plate in the presence of 5 μg/mL chloramphenicol at 26°C for 48 h. PCR was performed to confirm the colony bearing the plasmid integrated into the chromosome of the *V. anguillarum* 775 strains, and then the colony was incubated in 5 mL of LB medium overnight at 26°C. After two transfers, 50 μL of culture was plated in the LB plate in the presence of 10% sucrose and incubated at 26°C for 48 h. Finally, the correct mutant was confirmed by PCR, designating the Δ*VaryhB* mutant and the Δ*Vafur*Δ*VaryhB* mutant, respectively. To complement the Δ*VaryhB* mutant, the *VaryhB* gene with its promoter region was ligated into SmaI/ApaI-digested pBBR1MCS-2-Cm to obtain the plasmid pLYJ232. The plasmid pLYJ232 was transferred into the Δ*VaryhB* mutant by conjugation.

### 2.6 Quantitative real-time PCR analysis

To compare gene expression levels among WT, Δ*VaryhB* mutant, and Δ*Vafur* mutant, different *V. anguillarum* 775 cells were cultured under limited or rich iron conditions twice and grown to the exponential phase (OD_600 nm_ value of 0.8) under their respective iron conditions for the qRT-PCR assay. In brief, 1 mL of culture was collected at 11,000 *g* at 4°C. The RNAprep Pure Cell/Bacteria Kit (TIANGEN, China) was used to extract total RNA according to the manufacturer’s instructions. After DNA elimination, the PrimeScript™ RT reagent kit with the gDNA Eraser (TaKaRa, Japan) was used to obtain cDNA. The housekeeping gene *mreB*, encoding an actin protein, was used as an internal control. The qRT-PCR reactions were carried out in a 20-μL volume with 10 μL of 2xSYBR^@^Premix Ex Taq™ II (TaKaRa, Japan), 1 μL of cDNA template, 8.4 μL of ddH_2_O, and 0.3 μL of each of the forward and reverse primers (10 μM) using the real-time PCR system Applied Biosystems 7500 (Thermo Fisher Scientific, USA). The relative expression levels were calculated using the comparative C^T^ method (2^-ΔΔCT^) (Livak and Schmittgen, 2001). Three independent experiments were carried out, and values were obtained from representative experiments in triplicate.

### 2.7 RNA sequencing by Illumina HiSeq

For RNA sequencing, Δ*VaryhB* cells were grown under rich and limited iron conditions to the exponential phase. After RNA extraction, the RNA quality and quantity were assessed by the Agilent RNA 6000 Nano Kit (Agilent, USA), and rRNA was further eliminated using the Ribo-Zero™ Magnetic Kit (Epicentre). Then fragmented mRNA was primed with random primers. When the first-strand cDNA was synthesized, the second-strand cDNA was obtained by adding DNA polymerase I, RNase H, dNTP, and buffer. After purification, end-repairing, and poly(A)-tailing, fragments were ligated to Illumina sequencing adapters, and ones with a length of 300–500 bp were selected. Illumina HiSeq™ 4000 (Illumina, USA) was used for sequencing, and the collected data were analyzed by Shanghai Personal Biotechnology Co. Ltd. (Shanghai, China). Three independent samples under limited or rich iron conditions were used for RNA sequencing. The raw data from transcriptomic analysis have been submitted to the National Center for Biotechnology Information (NCBI) Sequence Read Archive database (accession No. PRJNA1186938). To identify genes showing different regulation in the WT and Δ*VaryhB* mutant, the raw data of the WT RNA-sequencing was obtained from the NCBI with an accession number of PRJNA1140836 (Li et al., 2024).

### 2.8 Siderophore assays

The CAS liquid assay was performed to calculate the production of siderophore as previously described (Li et al., 2024). In brief, different *V. anguillarum* 775 cells were grown in MM9 medium (0.3 g KH_2_PO_4_, 1 g NaCl, 1 g NH_4_Cl per L) in the presence of 0.2% casamino acid and 100 mM PIPES overnight at 26°C. After centrifugation, 500 μL of supernatant mixed with 500 μL of CAS assay solution (150 μM CAS, 15 μM FeCl_3_, 0.6 mM HDTMA, 500 mM piperazine buffer), 10 μL of 0.2 M 5-sulfosalicylic acid was supplemented, and the mixture was incubated for 5 min at room temperature. When siderophore is present, the siderophore could remove iron from the complex and lead to a decrease in the blue color of the mixture. The absorbance at 630 nm (A_630_) was examined. Siderophore units were calculated as follows:


$$\text{Siderophore}\;\text{unit}\;\left(\%\right)=\left[\frac{\text{Ar}-\text{As}}{\text{Ar}}\right]*100$$

A_r_ indicates the absorbance of the MM9 medium plus the CAS assay solution plus 5-sulfosalicylic acid; A_s_ indicates the absorbance of the tested sample. Three independent biological experiments were carried out, and values were obtained from representative experiments in triplicate.

### 2.9 Swimming motility assays

M9 swimming plates (M9 high salt broth with 0.3% agar supplemented with 50 μM 2, 2’-dipyridine or 50 μM FeCl_3_) were prepared and air-dried overnight at room temperature. Exponential phase *V. anguillarum* 775 cells were inoculated with a sterile toothpick on the plates at 26°C for 24 h, and the diameter of the “colony” was measured. Three independent experiments were performed, and values were calculated from representative experiments in triplicate.

### 2.10 Intracellular reactive oxygen species assays

The intracellular ROS levels were measured as described by Pasqua et al (Pasqua et al., 2017). with slight modifications. In brief, different *V. anguillarum* 775 strains were grown under different conditions to reach the exponential phase. Then, bacteria from 1 mL of the culture were harvested by centrifugation at 3,500 *g* at 4°C for 15 min. After being washed twice with phosphate-buffered saline (PBS), the cells were resuspended in PBS to reach an OD_600 nm_ value of 2.0, and 10 μM of DMSO-diluted H_2_DCFDA was supplemented. The mixture was incubated in the dark for 60 min at 30°C and washed twice with 1 mL of PBS. Finally, cells were resuspended in 200 μL of PBS for the fluorescence measurement (excitation: 485 nm; emission: 535 nm) using an Infinite 200 PRO microplate reader (TECAN, Switzerland). The assays were performed in three independent experiments, and values were obtained from representative experiments in triplicate.

### 2.11 Virulence assays

The infection assays were carried out on turbot as previously described (Li et al., 2024). In brief, turbot larvae (~20 g per fish) were intraperitoneally injected with 100 μL of a bacterial suspension (~1,000 CFU), and 100 μL of PBS was used as a control. Before injection, different *V. anguillarum* 775 strains were grown under limited iron conditions at 26°C to exponential phase, washed twice with PBS, and diluted in PBS. After bacterial injection, the turbots were incubated in fresh, filtered seawater at 20°C and observed daily for dead fish. Then gut bacteria of the dead fish were isolated, and mortalities were considered to result from *V. anguillarum* 775 strains only when the *V. anguillarum* 775 strain was found in pure culture (Crosa et al., 1977). Virulence was calculated by recording the number of survivors for 10 days post-injection. The assay was performed in three independent biological experiments. To examine the bacterial survival in turbot larvae, after 20 h of infection, spleens and livers were aseptically collected in PBS. After dilution, the supernatant was plated on M9 high salt plates, and the number of bacteria in the liver and the spleen was counted, which was shown as CFU/g. These turbot experiments were performed in accordance with the ethical guidelines of Shandong University.

### 2.12 Statistical analysis

If not specified, the Student *t* test (two-tailed) was used for statistical analysis in Microsoft Excel (Office 2021; Microsoft, Redmond, WA, USA). The statistical analysis of the survival rate was performed using the paired *t* test in GraphPad Prism 8.0.2 (GraphPad, San Diego, California).

## 3 Results

### 3.1 Identification of the *VaryhB* gene in *V. anguillarum* 775

**Figure 1:**
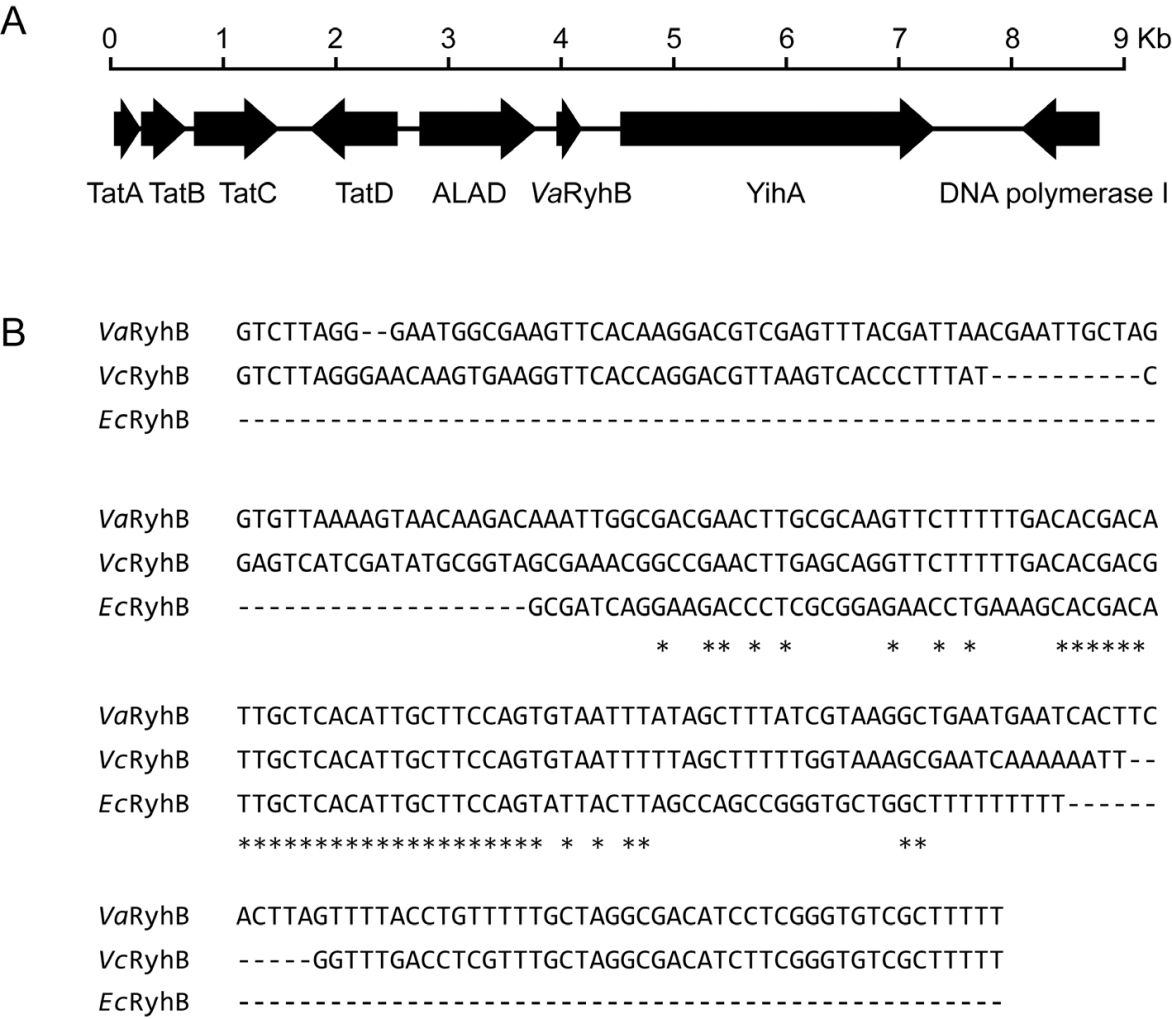
Identification of *Va*RyhB in *V. anguillarum* 775. **(A)** Organization map of the *VaryhB* gene in the genome of *V. anguillarum* 775. The *tatABCD* cluster is involved in the twin-arginine targeting (Tat) protein secretion system; *yihA* is involved in ribosome biogenesis; and delta-aminolevulinic acid dehydratase (ALAD) is responsible for heme biosynthesis. **(B)** RhyB sRNA alignment. The *ryhB* sequences from *V. anguillarum* 775 (*Va*), *V. cholerae* N16961 (*Vc*), and *E. coli* K-12 substr. MG1655. Sequences conserved in three strains are indicated with an asterisk (*).

A homolog of the *ryhB* gene, named *VaryhB*, was identified in the genome of *V. anguillarum* 775. As shown in **Figure 1A**, the sRNA *VaryhB* gene contains 228 bp and is located in the 734-bp intergenic region between VAA_RS01400, encoding a delta-aminolevulinic acid dehydratase (ALAD), and VAA_RS01405, encoding a YihA family ribosome biogenesis GTP-binding protein. Sequence alignment showed that *VaryhB* is relatively conserved with that in *V. cholerae*. Compared to *E. coli* RyhB (*Ec*RyhB), RyhB sRNAs from *V. anguillarum* and *V. cholerae* are much longer in the 5’ region, whereas these sRNAs harbor a conserved central region with the *E. coli Ec*RyhB (**Figure 1B**). The additional 5’ region in the *V. cholerae* RyhB (*Vc*RyhB) is proposed to be responsible for the stability of the sRNA structure (Mey et al., 2005). The longer RyhB sRNAs appear to be the major form among the *Vibrionaceae* (Davis et al., 2005; Mey et al., 2005). The longest RyhB is from *Vibrio parahaemolyticus*, with a length of 233 bp. However, it is unclear why longer RyhB sRNAs occur in the *Vibrionaceae*.

### 3.2 Identification of the *Va*RyhB regulon

**Figure 2:**
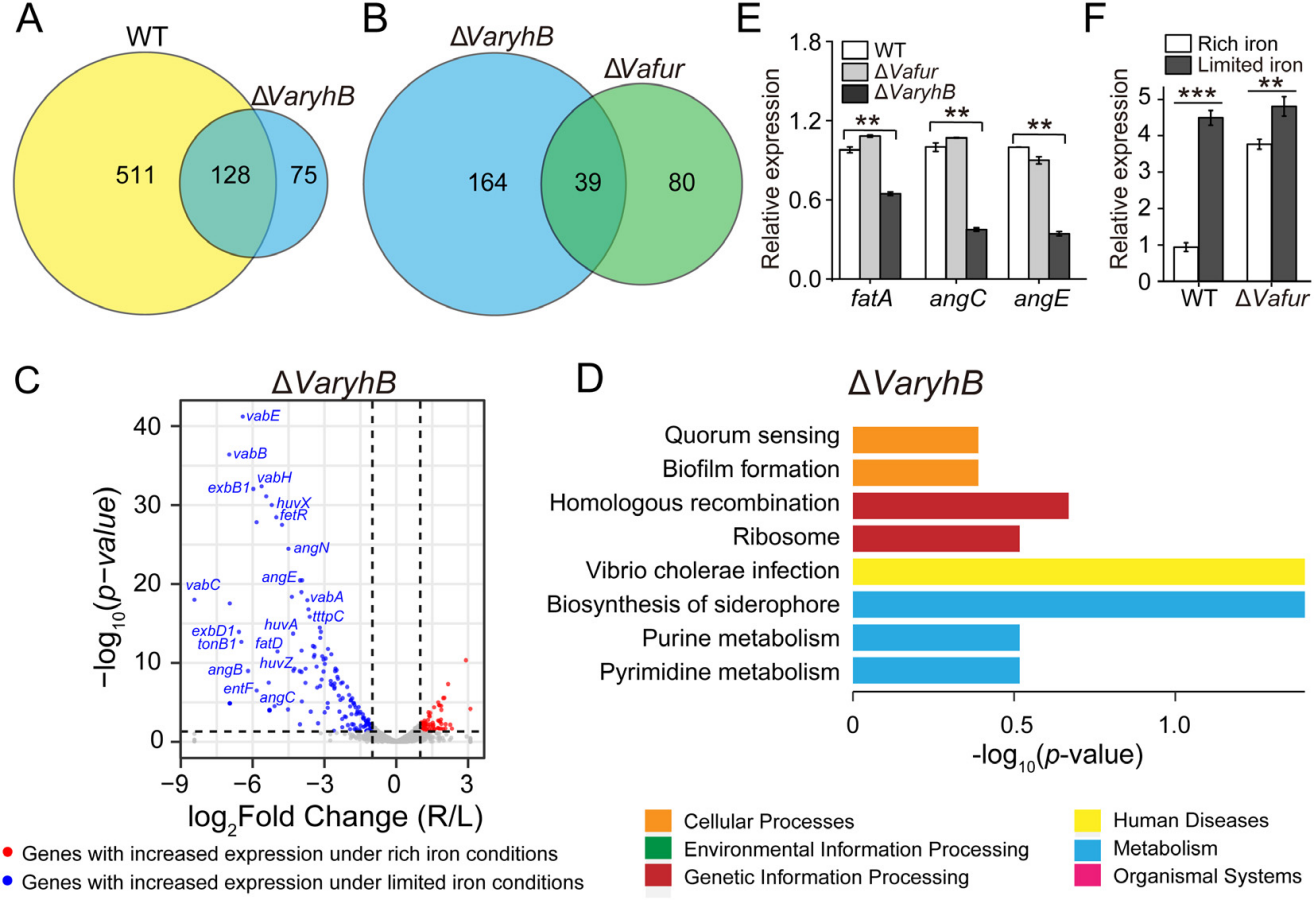
Transcriptomic analysis of the Δ*VaryhB* mutant under rich (R) and limited (L) iron conditions. **(A)** Comparison of iron-regulated genes in the WT and the Δ*VaryhB* mutant. In the WT, 639 genes displayed different expression levels under rich and limited iron conditions. In the Δ*VaryhB* mutant, 203 genes showed different expression levels under rich and limited iron conditions. **(B)** Comparison of iron-regulated genes in the Δ*VaryhB* mutant and the Δ*Vafur* mutant. In the Δ*Vafur* mutant, 119 genes showed different expression levels under rich and limited iron conditions. **(C)** Volcano plot showing iron-upregulated and iron-downregulated genes in the Δ*VaryhB* mutant. Iron-upregulated genes showed higher expression levels under rich iron conditions, and iron-downregulated genes showed higher expression levels under limited iron conditions. **(D)** COG enrichment analysis of iron-regulated genes in the Δ*VaryhB* mutant. The volcano plot and the COG enrichment of the WT cells are shown in **Supplementary Figure S1**, published by Li et al (Li et al., 2024). **(E)** Expression of genes *fatA*, *angC*, and *angE* in WT strain, Δ*Vafur* mutant, and Δ*VaryhB* mutant under limited iron conditions by qRT-PCR analysis. The expression values of different genes are shown as fold changes relative to their expression levels in the WT under limited iron conditions. **(F)** Expression of *VaryhB* gene in the WT and the Δ*Vafur* mutant under different iron conditions. Rich and limited iron conditions were obtained by adding 50 μM respective FeCl_3_ and 2, 2’-dipyridine into M9 high salt media. Results from representative experiments were obtained in triplicate, and values are indicated as means ± standard deviations. **, *p* < 0.01; ***, *p* < 0.001.

To understand the function of *Va*RyhB on iron homeostasis, a Δ*VaryhB* mutant was constructed, and RNA-seq-based transcription analysis was performed under different iron conditions. Compared to the WT cells (639 regulated genes) (Li et al., 2024), much fewer genes (203 genes) were regulated by iron in the Δ*VaryhB* mutant (**Figure 2A**): 139 genes had increased expression and 64 genes had decreased expression under limited iron conditions (*p* < 0.05 and |log_2_ fold change| ≥ 1, **Figure 2C**). However, compared to the Δ*Vafur* mutant (119 regulated genes) (Li et al., 2024), more regulated genes were observed in the Δ*VaryhB* mutant (**Figure 2B**). Most significantly up-regulated genes under limited iron conditions are associated with iron uptake (**Figure 2C**), which is similar to that in the WT. Clusters of orthologous groups of proteins (COG) analysis suggested that fewer categories were regulated by iron in the Δ*VaryhB* mutant compared to those in the WT. For example, compared to the WT (**Supplementary Figure S1**), flagellar assembly, sulfur relay system, and the TCA cycle were not differently expressed in the Δ*VaryhB* mutant under different iron conditions (**Figure 2D**). However, different regulation modes occurred among these pathways. Compared to the WT, genes involved in the TCA cycle in the Δ*VaryhB* mutant exhibited higher expression levels mainly under rich iron conditions; genes involved in the Fe-S assembly showed higher expression levels under both rich and limited iron conditions; while genes for chemotaxis and motility displayed lower expression levels under both rich and limited iron conditions (**Table 1**). Similar RyhB-mediated expression modes were observed in some microorganisms, such as *E. coli* (Massé and Gottesman, 2002; Desnoyers et al., 2009) and *V. cholerae* (Mey et al., 2005). Genes involved in denitrification were up-regulated by *Va*RyhB, and when *VaryhB* was absent, their expression levels were significantly decreased under rich and limited iron conditions. This is different from the expression mode of the *nap* operon in *E. coli* (Wang et al., 2015), which was down-regulated by RyhB. Most genes for iron uptake displayed similar regulation modes in the Δ*VaryhB* mutant and the WT strain and showed higher expression levels when iron was depleted. Several genes, including *angC*, *angE*, and *fatA*, appear to be activated by *V*aRyhB, and reduced expression levels were observed when *VaryhB* was deleted (**Table 1**). This activation may be important for bacterial growth in the host and thereby for pathogenesis. Consistent with this, qRT-PCR data suggested that the expression levels of *angC*, *angE*, and *fatA* were significantly reduced compared to those in the WT and the Δ*Vafur* mutant (**Figure 2E**). Differently, *angT* for iron release from the ferric-anguibactin complex and *exbB1* and *tonB1* for energy transmission for anguibactin and heme uptake were negatively regulated by *Va*RyhB. The expression mode of the *VaryhB* gene was also investigated by qRT-PCR. As shown in **Figure 2F**, the expression of the *VaryhB* gene was significantly repressed by iron in the WT. In the Δ*Vafur* mutant, the level of inhibition in response to rich iron conditions obviously became lower, indicating that the expression of the *VaryhB* gene is regulated by iron and *Va*Fur. Two different regulation modes occurred for genes involved in oxidative stress. In addition, genes responsible for T6SS were negatively regulated by *Va*RyhB, whereas genes for molybdenum cofactor (Moco) biosynthesis were positively regulated by *Va*RyhB. These two pathways of T6SS and Moco biosynthesis were first observed to be regulated by RyhB. Taken together, *Va*RyhB is involved in the regulation of genes involved in the TCA cycle, denitrification, Fe-S assembly, anguibactin-mediated iron uptake, oxidative resistance, and some virulence factors (motility and chemotaxis, and T6SS).

**Table 1:** Different expressed genes in Δ*VaryhB* mutant compared to the wild-type strain.

Gene	GenBank number	Description	Limited iron conditions		Rich iron conditions		Regulation
			^1^log_2_ fold change (WT/Δ*VaryhB*)	*p* value	^2^ log_2_ fold change (WT/Δ*VaryhB*)	*p* value	
TCA cycle							
*frdC*	VAA_RS02600	Fumarate reductase	-0.01	0.906	**-1.51**	0.024	Negative
*frdD*	VAA_RS02605	Fumarate reductase	-0.43	0.183	**-1.31**	0.045	Negative
*susB*	VAA_RS05360	α-ketoglutarate dehydrogenase	-0.12	0.560	**-2.32**	0.004	Negative
*susC*	VAA_RS05365	α-ketoglutarate dehydrogenase	0.95	0.066	**-1.70**	0.0002	Negative
*susD*	VAA_RS05370	Succinate-CoA synthetase	-0.12	0.640	**-2.54**	0.0005	Negative
*sdhC*	VAA_RS05335	Succinate dehydrogenase	-0.71	0.059	**-1.05**	0.025	Negative
*sdhA*	VAA_RS05345	Succinate dehydrogenase	-0.74	0.026	**-1.00**	0.037	Negative
Fe-S assembly							
*hscA*	VAA_RS11355	Fe-S cluster biosynthesis chaperone	**-1.67**	0.0009	-**1.04**	0.0004	Negative
*hscB*	VAA_RS11360	Fe-S cluster biosynthesis chaperone	**-1.21**	0.011	-0.50	0.147	Negative
*iscA*	VAA_RS11365	Fe-S cluster biosynthesis machinery	**-1.74**	0.0005	-**1.07**	0.011	Negative
*iscU*	VAA_RS11370	Fe-S cluster biosynthesis machinery	**-3.98**	0.010	**-3.44**	0.001	Negative
*iscS*	VAA_RS11375	Fe-S cluster biosynthesis machinery	**-1.72**	0.009	**-1.08**	0.010	Negative
*iscR*	VAA_RS11380	Fe-S cluster biosynthesis machinery	**-2.05**	0.011	-1.28	0.070	Negative
Chemotaxis and motility							
*fliM*	VAA_RS05180	Flagella machinery	**1.26**	0.0002	**1.27**	0.0008	Positive
*fliO*	VAA_RS05190	Flagella machinery	**2.02**	0.0002	**1.23**	0.0002	Positive
*fliP*	VAA_RS05195	Flagella machinery	**1.97**	0.0008	**2.00**	0.0005	Positive
*fliQ*	VAA_RS05200	Flagella machinery	**1.48**	0.018	**1.68**	0.0001	Positive
*fliR*	VAA_RS05205	Flagella machinery	**1.39**	0.0002	**1.99**	0.0002	Positive
*cheY*	VAA_RS05455	Chemotaxis	**1.41**	0.027	**1.01**	0.002	Positive
*cheW*	VAA_RS05485	Chemotaxis	**2.04**	0.0003	**1.50**	0.002	Positive
*cheW*	VAA_RS07520	Chemotaxis	**1.64**	0.0001	**1.29**	0.0001	Positive
*cheR*	VAA_RS07545	Chemotaxis	**2.26**	0.009	**1.97**	0.0008	Positive
Denitrification							
*napC*	VAA_RS17925	Periplasmic nitrate reductase subunit C	0.98	0.035	**1.79**	0.010	Positive
*napB*	VAA_RS17930	Periplasmic nitrate reductase subunit B	0.97	0.096	**2.26**	0.016	Positive
*napA*	VAA_RS17935	Periplasmic nitrate reductase subunit A	**1.39**	0.020	**2.16**	0.035	Positive
*napD*	VAA_RS17940	Periplasmic nitrate reductase subunit D	**2.07**	0.005	**2.52**	0.00004	Positive
*napF*	VAA_RS17945	Periplasmic nitrate reductase subunit F	0.66	0.4010	**2.67**	0.0002	Positive
Fe uptake							
*angC*	gb|AAO07758.1|	Anguibactin biosynthesis	**1.82**	0.014	0.03	0.886	Positive
*angE*	gb|AAF93937.1|	Anguibactin biosynthesis	**1.26**	0.042	0.16	0.178	Positive
*fatA*	gb|AAA91581.1|	Anguibactin transport	**1.47**	0.022	0.70	0.011	Positive
*angT*	gb|AAA79861.1|	Iron release from ferric-anguibactin	**-1.50**	0.026	**-1.72**	0.012	Negative
*exbB1*	VAA_RS07405	Energy transmission for heme uptake	**-2.08**	0.021	-0.66	0.228	Negative
*tonB1*	VAA_RS07410	Energy transmission for heme uptake	**-1.25**	0.003	-0.60	0.140	Negative
Oxidative stress							
*prxQ1*	VAA_RS05025	Thioredoxin peroxidase	0.70	0.020	**2.18**	0.0001	Positive
*prx5*	VAA_RS02690	Peroxiredoxin	0.39	0.223	**2.43**	0.00005	Positive
*msrA*	VAA_RS03150	Peptide methionine sulfoxide reductase	**-1.61**	0.001	**-2.29**	0.00002	Negative
*prxQ2*	VAA_RS11445	Thioredoxin peroxidase	-0.21	0.300	**-1.15**	0.039	Negative
Type VI secretion system							
*vtsA1*	VAA_RS07110	Cap protein	-0.37	0.451	**-1.86**	0.039	Negative
*vtsB1*	VAA_RS07115	Sheath protein	-0.33	0.242	**-1.11**	0.022	Negative
*vtsC1*	VAA_RS07120	Sheath protein	0.48	0.109	**-1.39**	0.001	Negative
*vtsD1*	VAA_RS07125	Hexameric ring	-0.13	0.607	**-1.74**	0.003	Negative
*vtsE1*	VAA_RS07130	Baseplate	0.30	0.452	**-1.36**	0.003	Negative
*vtsG1*	VAA_RS07140	Baseplate	**-2.23**	0.009	**-3.72**	0.0008	Negative
*vtsI1*	VAA_RS07155	Hub protein	0.62	0.214	**-1.95**	0.006	Negative
*vtsJ1*	VAA_RS07160	Membrane Complex	-0.55	0.013	**-2.49**	0.040	Negative
*vtsL1*	VAA_RS07170	Membrane Complex	0.14	0.640	**-1.98**	0.008373	Negative
*vtsM1*	VAA_RS07175	Membrane Complex	0.39	0.169	**-1.44**	0.102988	Negative
*vtsN1*	VAA_RS07180	T6SS-associated FHA protein	-0.35	0.323	**-2.83**	0.035833	Negative
*tssJ*	VAA_RS07185	Membrane Complex	-0.27	0.268	**-2.02**	0.01953	Negative
Molybdenum cofactor (Moco) biosynthesis							
*moaE*	VAA_RS10095	Moco biosynthesis unitE	-0.21	0.159	**1.50**	0.003	positive
*moaC*	VAA_RS10105	Moco biosynthesis unitC	-0.02	0.781	**2.40**	0.002	positive
*moaB*	VAA_RS10110	Moco biosynthesis unitB	0.09	0.641	**2.23**	0.006	positive
*moaA*	VAA_RS10115	Moco biosynthesis unitA	-0.47	0.301	**1.51**	0.0006	positive

**^1^** The value of the log_2_ fold change was obtained by calculating the gene expression levels in the WT divided by those in the Δ*VaryhB* mutant under limited iron conditions.

**^2^** The value of the log_2_ fold change was obtained by calculating the gene expression levels in the WT divided by those in the Δ*VaryhB* mutant under rich iron conditions.

The bold values indicate genes showing different expression levels in the WT and the Δ*VaryhB* mutant.

### 3.3 Absence of *VaryhB* leads to impaired growth under rich and limited iron conditions

**Figure 3:**
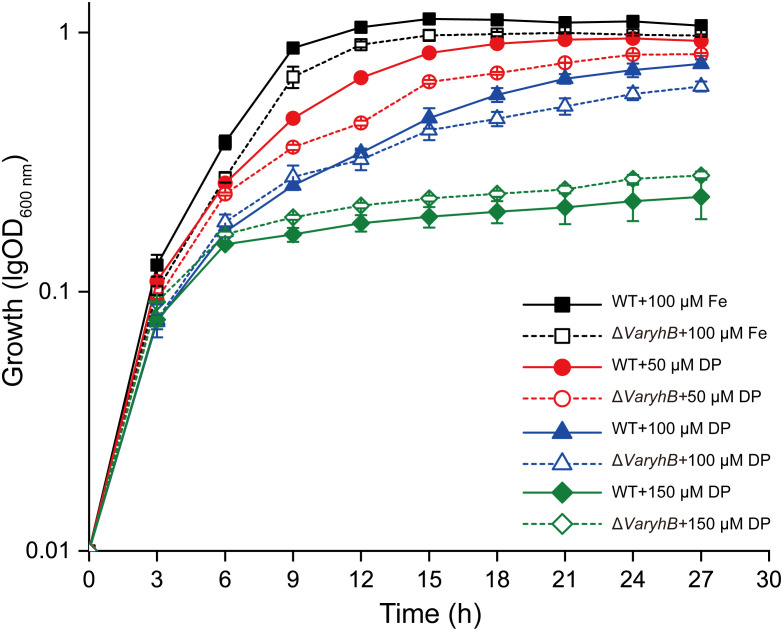
Growth of WT and the Δ*VaryhB* strain under different iron conditions. Results from representative experiments were obtained in triplicate, and the values are shown as means ± standard deviations. Fe, FeCl_3_; DP, 2, 2’-dipyridine.

**Figure 4:**
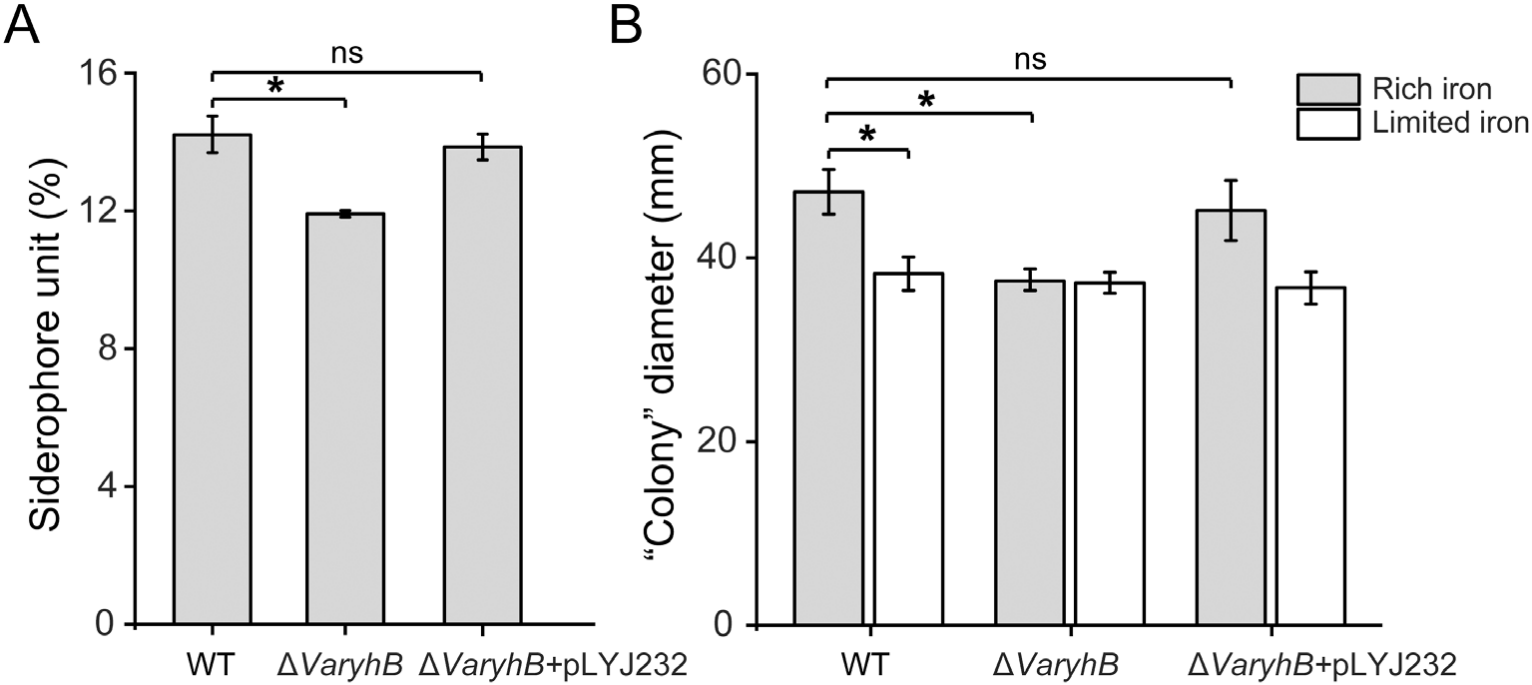
Siderophore synthesis and motility comparison in the WT, the Δ*VaryhB* mutant, and the Δ*VaryhB* complementation strain (Δ*VaryhB*+pLYJ232). **(A)** Siderophore production quantified by CAS liquid assay. **(B)** Swimming motility ability examined as “colony” diameter (mm). Rich and limited iron conditions were obtained by adding 50 μM respective FeCl_3_ and 2, 2’-dipyridine into M9 high salt media. Results from representative experiments were obtained in triplicate, and values are shown as means ± standard deviations. *, *p* < 0.05; ns, no significance.

Since the abnormal iron regulation in the Δ*VaryhB* mutant may cause impaired growth, the growth of the Δ*VaryhB* mutant strain was first examined under different iron conditions. When grown under iron-rich conditions, the Δ*VaryhB* mutant exhibited slightly decreased growth (**Figure 3**), similar to that of the Δ*Vafur* mutant. However, different from that of the Δ*Vafur* mutant (an increased growth yield), when grown under iron-poor conditions, the Δ*VaryhB* mutant strain showed attenuated growth. The impaired growth under limited iron conditions may be caused by reduced expression of genes for siderophore synthesis and transport (**Table 1**). In line with this, the siderophore content was slightly reduced in the Δ*VaryhB* mutant compared to that in the WT (**Figure 4A**). This abnormal growth in both poor and rich iron conditions was also observed in the *E. coli* Δ*ryhB* mutant (Jacques et al., 2006). The complementation of the Δ*VaryhB* mutant restored the WT-like growth in the presence of 50 μM 2, 2’-dipyridine (**Supplementary Figure S2**). Our data indicated that worse multiplication likely occurs during the Δ*VaryhB* infection, thereby leading to reduced virulence in the Δ*VaryhB* mutant strain.

### 3.4 Role of *Va*RyhB in swimming motility

Since genes for motility in the Δ*VaryhB* mutant exhibited reduced expression levels, the swimming motility was further tested under different iron conditions. As shown in **Figure 4B**, no iron-dependent regulation occurred when *VaryhB* was absent, and the capability of swimming motility in the Δ*VaryhB* mutant under rich and limited iron conditions was similar to that of the WT under limited iron conditions. Therefore, *Va*RyhB is essential to iron-regulated swimming behavior. Notably, the swimming motility of the *VaryhB* mutant was much greater than that of the *Vafur* mutant under limited and rich iron conditions (Li et al., 2024), although a similar phenotype of no iron-dependent regulation was observed in the two mutants.

### 3.5 Defective oxidative resistance in *Va*Fur-depleted cells is caused by *Va*RyhB

**Figure 5:**
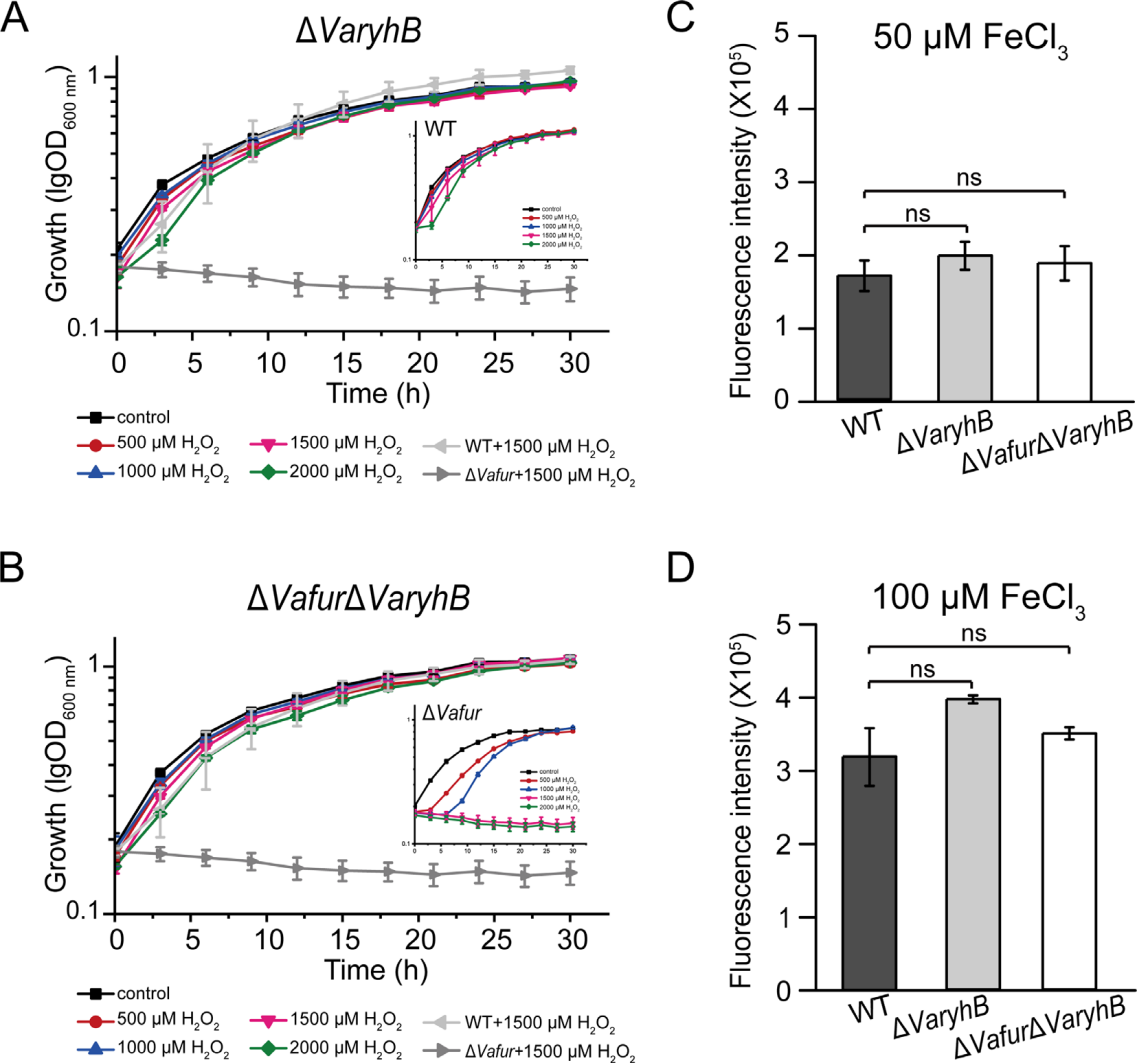
Oxidative-sensitive analysis of Δ*VaryhB* mutant and Δ*Vafur*Δ*VaryhB* mutant. **(A)** Growth curve of the Δ*VaryhB* mutant under rich iron conditions (50 μM FeCl_3_) in the presence of different concentrations of H_2_O_2._ **(B)** Growth of the Δ*Vafur*Δ*VaryhB* mutant under rich iron conditions (50 μM FeCl_3_) in the presence of different concentrations of H_2_O_2_. **(C)** and **(D)** ROS detection using the fluorescence probe H_2_DCFDA. Cells were grown in the presence of 50 μM FeCl_3_ **(C)** or 100 μM FeCl_3_ **(D)** before H_2_DCFDA treatment. As a control, the growth of the WT and the Δ*Vafur* mutant was also shown in **(A)** and **(B)** which has been published recently (Li et al., 2024). Results from representative experiments were obtained in triplicate, and values are indicated as means ± standard deviations. ns, no significance.

In many organisms, such as *E. coli*, the expression of the superoxide dismutase gene *sodB* was indirectly regulated by Fur through the RyhB (Massé and Gottesman, 2002; Troxell and Hassan, 2013). On the other hand, in *V. anguillarum* 775, the Δ*Vafur* mutant is much more sensitive to hydrogen peroxide than the WT strain (Li et al., 2024). Therefore, the impaired oxidative resistance in the Δ*Vafur* mutant may be caused by *Va*RyhB. To verify this, the sensitivity of the Δ*VaryhB* mutant to H_2_O_2_ was examined. As shown in **Figure 5A**, the presence of H_2_O_2_ did not affect the growth of Δ*VaryhB* mutant under rich iron conditions. In addition, the intercellular ROS levels were also tested using the ROS reactive probe H_2_DCFDA (Pasqua et al., 2017). In line with the growth in the presence of H_2_O_2_, the intracellular ROS levels in the Δ*VaryhB* mutant were similar to those of the WT in media with both 50 μM and 100 μM FeCl_3_ (**Figures 5B, D**). Moreover, a Δ*Vafur*Δ*VaryhB* double deletion mutant showed similar growth and ROS levels with WT and Δ*VaryhB* mutant (**Figures 5B–D**), further demonstrating that the defective oxidative resistance in *Va*Fur-depleted cells results from the activated *Va*RyhB, the expression of which is repressed by *Va*Fur under rich iron conditions in the WT. Therefore, *Va*Fur in *V. anguillarum* 775 adopts a “RyhB-dependent” mechanism to defend against oxidative stress.

### 3.6 Absence of *VaryhB* attenuates the pathogenicity of *V. anguillarum* 775

**Figure 6:**
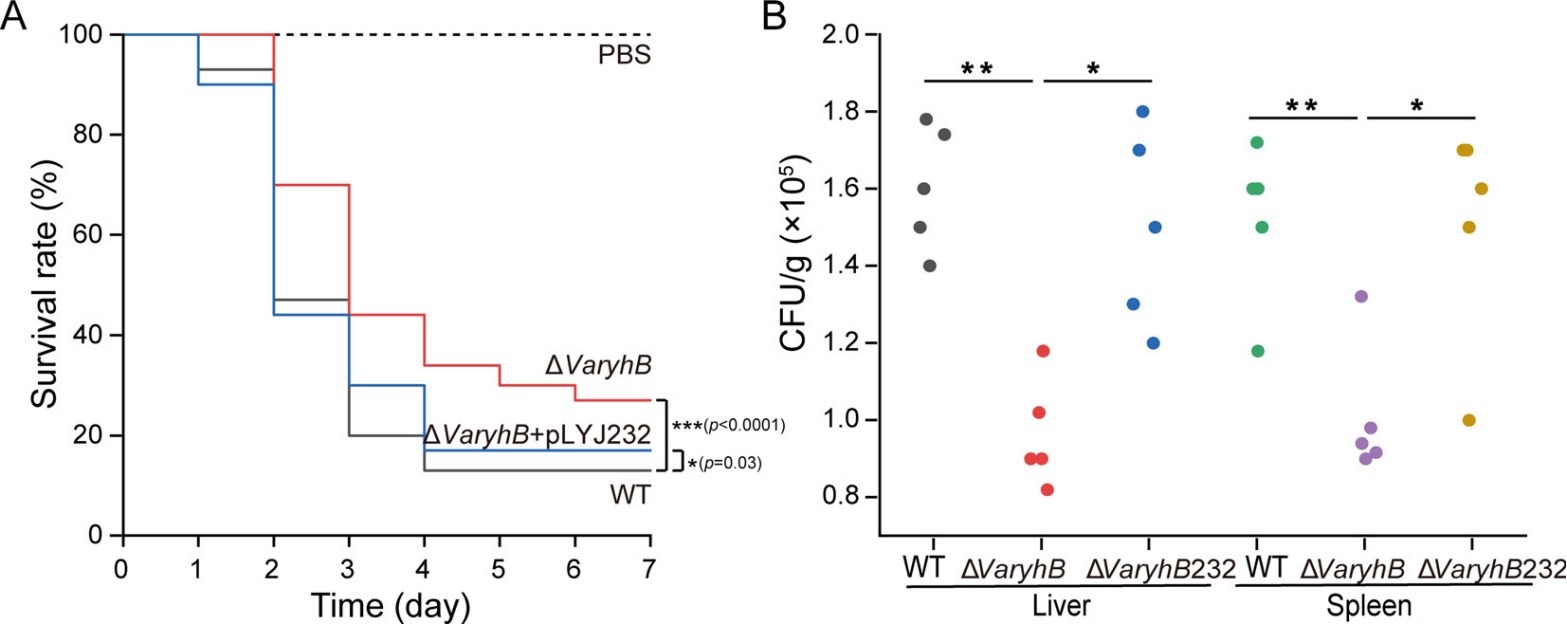
Virulence analysis of WT and the Δ*VaryhB* mutant. **(A)** Survival rate of turbot larvae infected by WT, the Δ*VaryhB* mutant, and the Δ*VaryhB* complementation strain (Δ*VaryhB*+pLYJ232). Fifteen turbot larvae were intraperitoneally inoculated with ~1,000 CFU of a bacterial suspension from a 12-h growing culture under limited iron conditions with PBS as a control. Fish survival was observed for up to 10 days, and no fish died after 6 days. Results were obtained from three independent experiments, and values are shown as means ± standard deviations. **(B)** Bacterial colonization in the liver and spleen. Livers and spleens were aseptically collected from five turbot larvae after 20 h of infection, and bacterial numbers were shown as CFU/g. One representative experiment was shown. Δ*VaryhB*232, the Δ*VaryhB* complementation strain. *, *p* < 0.05; **, *p* < 0.01; ***, *p* < 0.001.

RyhB in many pathogens is controlled by Fur and mainly functions under poor iron conditions (Chareyre and Mandin, 2018). On the other hand, a significantly reduced pathogenicity of the Δ*Vafur* mutant was observed in *V. anguillarum* 775 (Li et al., 2024). It was proposed that the decreased pathogenicity of the Δ*Vafur* mutant may be indirectly caused by RyhB. Therefore, to uncover the role of *Va*RyhB in the pathogenesis, turbot larvae were intraperitoneally challenged using the WT strain and the Δ*VaryhB* mutant, respectively. Survival was observed up to 10 days post-infection, and no turbot larvae died in the control group. In the WT infection group, 90% of turbot larvae died after 4 days (**Figure 6A**), whereas deletion of *VaryhB* resulted in a 20% reduction in the lethality rate compared to that of the WT infection group. When infected by the Δ*VaryhB* complementation strain, although the pathogenicity was greater than that of the Δ*VaryhB* mutant, the final lethality rate was lower than that of the WT strain. This may be caused by the loss of the complementation plasmid pLYJ232 in the host. In agreement with this, the Δ*VaryhB* population in the liver and spleen of turbot larvae was also decreased compared to that of the WT strain (**Figure 6B**). The complementation plasmid, pLYJ232, could restore bacterial numbers in the liver and spleen of turbot larvae back to the WT-like level. These data indicated that *Va*RyhB in *V. anguillarum* 775 is required for pathogenicity. However, compared to the Δ*Vafur* mutant, which showed a higher than 50% survival rate (Li et al., 2024), *Va*RyhB has less effect on the *V. anguillarum* pathogenicity. These indicated that in the Δ*Vafur* mutant, the reduced pathogenicity may not be mostly caused by irregulated *Va*RyhB.

## 4 Discussion

Fur acts as a global transcriptional repressor to control iron homeostasis in many microorganisms. In certain cases, Fur can also activate gene expression via a sRNA RyhB-dependent model, first demonstrated by Massé and Gottesman in *E. coli* (Massé and Gottesman, 2002). Similar to observations in *E. coli* strains (Salvail et al., 2010; Porcheron et al., 2014) and *Klebsiella pneumoniae* (Huang et al., 2012), when iron was depleted, *Va*RyhB in *V. anguillarum* 775 could promote the expression of some genes involved in biosynthesis (*angC* and *angE*) and transport (*fatA*) of the siderophore, anguibactin. Therefore, the deletion of *VaryhB* resulted in impaired growth and decreased siderophore production under limited iron conditions, which may lead to reduced virulence. In *V*. *parahaemolyticus*, this *Va*RyhB-dependent upregulation is suggested to be due to the increased stability of the *Va*RyhB target, a polycistronic mRNA responsible for siderophore biosynthesis (Tanabe et al., 2013). The putative thioesterase gene, *angT*, which may be involved in the release of iron from the ferric-anguibactin complex (Wertheimer et al., 1999), was repressed by *Va*RyhB, whereas it was not regulated by *Va*Fur (Li et al., 2024). However, deletion of the *angT* gene only caused a decrease, but not a complete shutoff, of anguibactin production (Wertheimer et al., 1999). Alternatively, *angT* is also proposed to function on the anguibactin release from a pantothenate site (Wertheimer et al., 1999). In this case, reduced expression of *angT* is required to help the bacterium cope with iron scarcity.

In addition to certain genes for siderophore synthesis, the expression of genes for chemotaxis and motility is also promoted by *Va*RyhB. Loss of *Va*RyhB led to reduced motility capability under rich iron conditions, also observed in *E. coli* (Beauchene Nicole et al., 2015; Melamed et al., 2016). Since reduced motility was also observed in the Δ*Vafur* mutant, the regulation of motility genes in the Δ*Vafur* mutant is likely independent of *Va*RyhB. In *V. cholerae*, although RyhB positively regulates motility, the *ryhB* mutant showed decreased motility under limited iron conditions (Mey et al., 2005). In contrast, in *Salmonella typhimurium*, RyhB plays a negative role in the regulation of flagellar and chemotaxis genes, and increased motility was observed in the *ryhB* mutant (Kim and Kwon, 2013). Although the precise regulatory mechanisms are not fully understood, modulation of chemotaxis and motility may be essential for the cell to navigate toward optimal iron conditions.

Despite positive regulation, *Va*RyhB inhibited the expression of most targets, including genes for the TCA cycle, Fe-S assembly, and the T6SS system. Among these, the regulation mechanism of the TCA and Fe-S assembly has been extensively studied (Troxell and Hassan, 2013; Porcheron and Dozois, 2015; Chareyre and Mandin, 2018), with RyhB repressing these targets through binding to their mRNAs. Although it is well-established that Fur plays a crucial role in the expression of the VI secretion system, this is the first observation that RyhB represses the expression of T6SS genes. Furthermore, oxidative resistance experiments indicated *Va*RyhB-dependent regulation in *V. anguillarum* 775: under iron-replete conditions, the Δ*Vafur* mutant was more sensitive to H_2_O_2_, but deletion of *VaryhB* in the Δ*Vafur* mutant restored protection against H_2_O_2_ toxicity. However, two distinct regulatory modes were observed: upregulation and downregulation of genes involved in oxidative defense. Two peroxidase genes, *prxQ1* and *prx5*, displayed reduced expression in the Δ*VaryhB* mutant, similar to the Δ*Vafur* mutant (Li et al., 2024). Differently, gene *prxQ2* showed greater expression in the Δ*VaryhB* mutant under rich iron conditions but was not regulated by *Va*Fur. Therefore, expanded research on oxidative defense is required to fully elucidate the roles of different peroxidase genes in *V. anguillarum*.

## 5 Conclusion

In conclusion, our work revealed that in *V. anguillarum*, the sRNA *Va*RyhB plays an important role in the inhibition of genes involved in the TCA cycle, Fe-S assembly, and the type VI secretion system. In addition, it is essential for the activation of siderophore synthesis, chemotaxis and motility, and anaerobic denitrification. These regulation processes are not always related to a *Va*Fur regulatory pathway. Although iron is found to be the major signal for RyhB regulation, other environmental signals might also be present for RyhB to respond to variable environments. For example, RyhB in *E. coli* has different mRNA targets under aerobic and anaerobic conditions (Beauchene Nicole et al., 2015). Therefore, an expanded search of the *Va*RyhB signal will gain more insights into its function on bacterial survival in different habitats.

## Data Availability

The datasets presented in this study can be found in online repositories. The names of the repository/repositories and accession number(s) can be found in the article/**Supplementary Material**.
