# Supplementary material for: Characterization of the Vibrio anguillarum VaRyhB regulon and role in pathogenesis
Source: Front Cell Infect Microbiol. 2025 Jan 21;14:1531176. doi: 10.3389/fcimb.2024.1531176 (PMC11790442; doi:10.3389/fcimb.2024.1531176)
Supplement: Supplementary file 1 [file DataSheet1.docx]

Supplementary Material


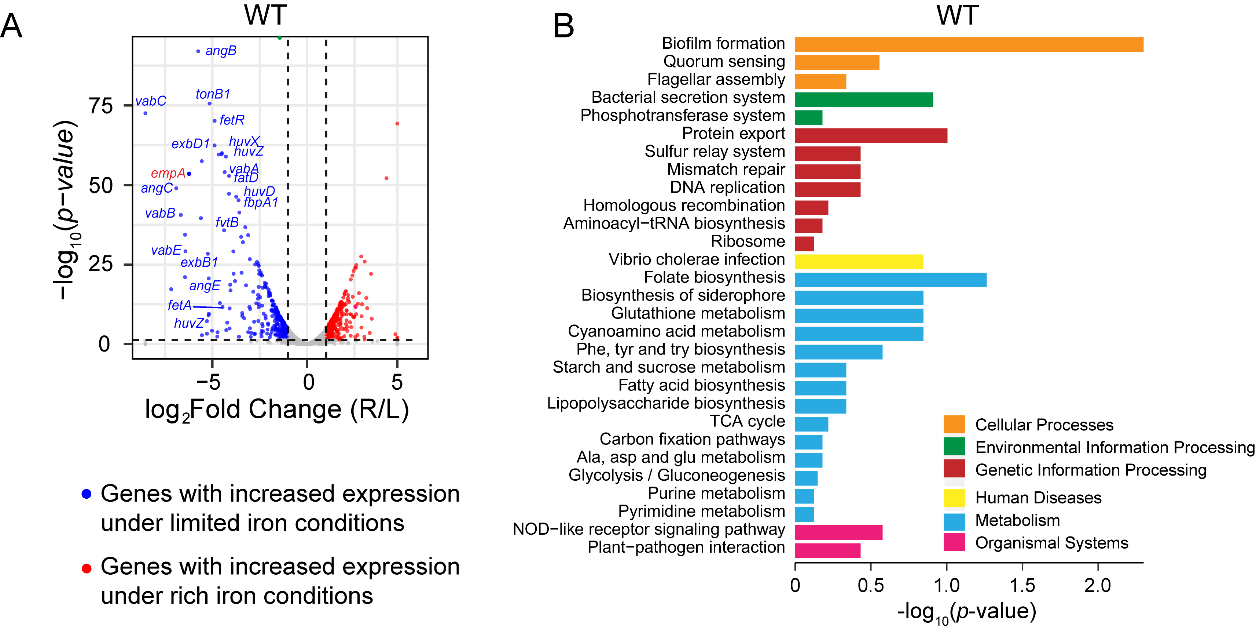


**Figure S1** Transcriptome analysis of the WT strain under limited and rich iron conditions. (A) Volcano plot showing iron-upregulated and iron-downregulated genes in the WT. (B) COG enrichment analysis of iron-regulated genes in the WT. These data have been published by Li et al. (Li et al., 2024).


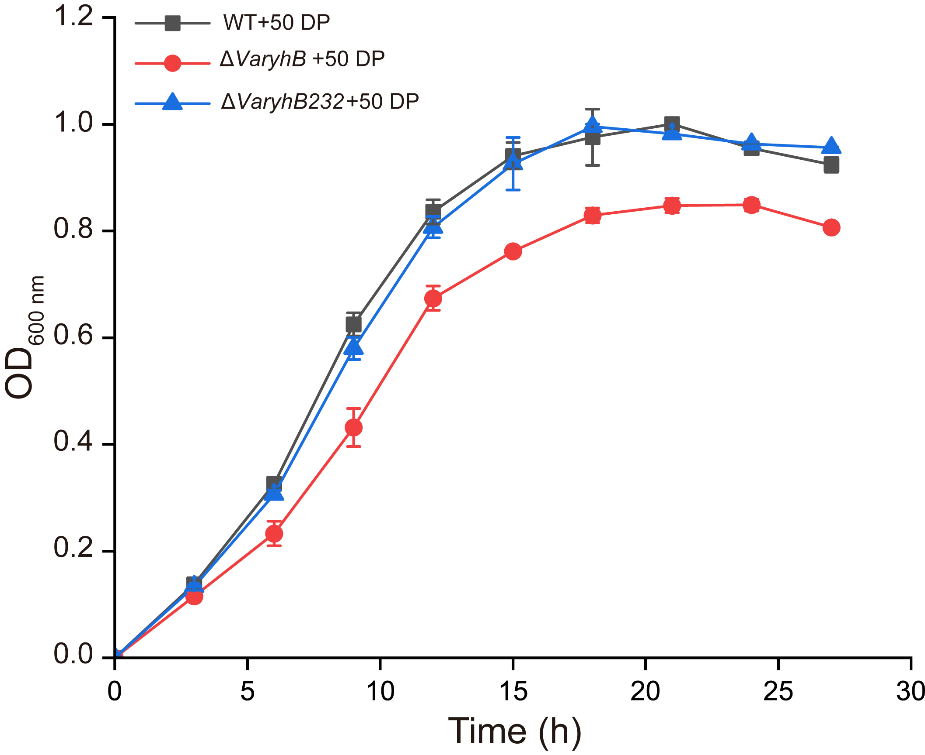


**Figure S2** Growth of WT, the Δ*VaryhB* strain, and the Δ*VaryhB* complementation strain (Δ*VaryhB*232) under limited iron conditions. Results from representative experiments were obtained in triplicate, and the values are shown as means ± standard deviations. 50 DP, 50 μM 2, 2’-dipyridine.

**Table S1 Strains and plasmids used in this study**

| **Strain or plasmid** | **Description** | **Source or reference** |
| --- | --- | --- |
| **Strains** |  |  |
| *Vibrio anguillarum* 775 (ATCC 68554) | Wild-type | Laboratory storage |
| *E. coli* CC118 | The strain used for the construction of deletion plasmids | (Cote et al., 2000) |
| *E. coli* X7213 | The donor strain for conjugation | (Roland et al., 1999) |
| Δ*Vafur* | *Vafur* deletion mutant in strain *V. anguillarum* 775 | (Li et al., 2024) |
| Δ*VaryhB* | *VaryhB* deletion mutant in strain *V. anguillarum* 775 | This study |
| Δ*Vafur*Δ*VaryhB* | *Vafur* and *VaryhB* double deletion mutant in strain *V. anguillarum* 775 | This study |
| Δ*VaryhB*+pLYJ232 | *VaryhB* complementation strain | This study |
| **Plasmids** |  |  |
| pRE112 | *oriT, oriV, sacB1,* Cm^r^, counterselectable suicide plasmid | (Edwards et al., 1998) |
| pBBR1MCS-2-Cm | Cm^r^, mobilizable broad-host-range vector | (Li et al., 2024) |
| pLYJ220 | pRE112 plus 2-kb fused flanking section of *VaryhB* | This study |
| pLYJ232 | pBBR1MCS-2-Cm plus *VaryhB* with its own promoter | This study |

**Table S2 Primers used in this study**

| **Primers** | **Sequence (5'-3')** | **Source or reference** |
| --- | --- | --- |
| **Construction of knockout plasmids** | |  |
| VaRyhB-F1 | GCTCTAGACGAAGAAGCGCTGTATTTGG | This study |
| VaRyhB-R1 | CTCTTGAAAATGAGCGATAAATGGTTAATAATAATGATTC | This study |
| VaRyhB-F2 | GAATCATTATTATTAACCATTTATCGCTCATTTTCAAGAG | This study |
| VaRyhB-R1 | TCCCCCGGGAGAGCCGCGAAAACCCAATT | This study |
| **Construction of complementation plasmids** | |  |
| *VaryhB*-F | TCCCCCGGGGTTGTGAATTTAGGCAGGGG | This study |
| *VaryhB*-R | GTACGGGCCCCTTTTATTCACAACAAAAGC | This study |
| **qRT-PCR** |  |  |
| *mreB*-qF | TTATTCTTCGCTAAATAGGTCGCCACC | (Li et al., 2024) |
| *mreB*-qR | CCGCCTCTTGACCGAAGAAA | (Li et al., 2024) |
| *fatA*-qF | GACTCATCAGGTAGCGACCT | This study |
| *fatA*-qR | GTGATACTGATGGGCAATGC | This study |
| *angC*-qF | ATACAGTGAGTAACTCATCTACTTG | This study |
| *angC*-qR | CATGAATCCTCACCTCAAAG | This study |
| *angE*-qF | GTGGTCATGAATAATCTTAACCCGACCG | This study |
| *angE*-qR | CGTCAGGGCTTTTTTTTACGGAATC | This study |
| *ryhB-*qF | CGATTAACGAATTGCTAGGT | This study |
| *ryhB*-qR | CAATGTGAGCAATGTCGTGT | This study |

**REFERENCES**

Cote, C.K., Cvitkovitch, D., Bleiweis, A.S., and Honeyman, A.L. (2000). A novel beta-glucoside-specific PTS locus from *Streptococcus mutans* that is not inhibited by glucose. *Microbiology (Reading)* 146 ( Pt 7)**,** 1555-1563. doi: 10.1099/00221287-146-7-1555.

Edwards, R.A., Keller, L.H., and Schifferli, D.M. (1998). Improved allelic exchange vectors and their use to analyze 987P fimbria gene expression. *Gene* 207(2)**,** 149-157. doi: 10.1016/s0378-1119(97)00619-7.

Li, Y., Yu, X., Li, P., Li, X., and Wang, L. (2024). Characterization of the ferric uptake regulator *Va*Fur regulon and its role in *Vibrio anguillarum* pathogenesis. *Applied and Environmental Microbiology* 0(0)**,** e01508-01524. doi: doi:10.1128/aem.01508-24.

Roland, K., Curtiss, R., 3rd, and Sizemore, D. (1999). Construction and evaluation of a delta *cya* delta *crp Salmonella typhimurium* strain expressing avian pathogenic *Escherichia coli* O78 LPS as a vaccine to prevent airsacculitis in chickens. *Avian Dis* 43(3)**,** 429-441.
